# Flecainide reduces exercise-induced arrhythmic burden in PKP2-related arrhythmogenic cardiomyopathy

**DOI:** 10.1093/europace/euag260

**Published:** 2026-09-16

**Authors:** Matteo Castrichini, Ramin Garmany, Michael J Ackerman, John R Giudicessi

**Affiliations:** Department of Cardiovascular Medicine, Mayo Clinic, 200 First Street SW, Rochester, MN 55905, USA; Mayo Clinic Medical Scientist Training Program, Mayo Clinic Graduate School of Biomedical Sciences, Mayo Clinic Alix School of Medicine, Rochester, MN, USA; Department of Molecular Pharmacology and Experimental Therapeutics, Mayo Clinic, 200 First Street SW, Rochester, MN 55905, USA; Department of Cardiovascular Medicine, Mayo Clinic, 200 First Street SW, Rochester, MN 55905, USA; Department of Molecular Pharmacology and Experimental Therapeutics, Mayo Clinic, 200 First Street SW, Rochester, MN 55905, USA; Department of Pediatrics (Division of Pediatric Cardiology), Mayo Clinic, Rochester, MN, USA; Department of Cardiovascular Medicine, Mayo Clinic, 200 First Street SW, Rochester, MN 55905, USA; Department of Molecular Pharmacology and Experimental Therapeutics, Mayo Clinic, 200 First Street SW, Rochester, MN 55905, USA

**Keywords:** Arrhythmogenic cardiomyopathy, Exercise stress testing, Flecainide, PKP2, Risk-stratification, Ventricular arrhythmias

Exercise-induced ventricular arrhythmias are a well-recognized feature of arrhythmogenic cardiomyopathy (ACM), particularly among individuals with pathogenic/likely pathogenic (P/LP) variants in the *PKP2*-encoded plakophilin-2.^1^ Recent data suggest that PKP2-ACM patients have an increased burden of complex ventricular ectopy/non-sustained ventricular tachycardia during the recovery phase of exercise stress testing (EST) that may correlate with future risk of sustained ventricular arrhythmias (VA).^2^

Although the use of serial ESTs to monitor therapeutic efficacy and guide treatment intensification is well established for catecholaminergic polymorphic ventricular tachycardia (CPVT),^3,4^ whether a similar approach could be utilized for PKP2-ACM, particularly given the efficacy and increased utilization of flecainide therapy in ACM^5^ remains to be seen. Therefore, we sought to evaluate whether flecainide attenuates exercise and recovery-phase arrhythmias in PKP2-ACM.

Retrospective analysis of 1049 genotype-positive ACM patients was used to identify those with P/LP variants in *PKP2* that were started on flecainide with available EST data. Clinical variables, medication history, and arrhythmia data were collected from electronic records and EST reports. Arrhythmias were assessed during the baseline and peak of the exercise (non-recovery phases) and EST recovery phase. Arrhythmic burden was measured using the frequency of premature ventricular contractions (PVCs) and PVCs/min, couplets, bigeminy, non-sustained ventricular tachycardia (NSVT), sustained VT, and ventricular fibrillation (VF), and compared between the EST performed pre- and post-initiation of flecainide. PVCs per minute were calculated as the total number of ventricular ectopic beats, including isolated PVCs, couplets, and all beats within runs of NSVT or VT, divided by the duration (in min) of the corresponding EST phase.

Overall, 147/1049 (14%) of patients had a P/LP variant in *PKP2*, with 89/147 (60%) having EST data. Amongst these, 11/89 (13%) underwent at least one EST while receiving flecainide, for a total of 35 on-flecainide ESTs. Eight of these patients had undergone both on and off flecainide for a total of 29 ESTs, allowing for comparison of arrhythmic burden on and off flecainide.

The eight patients included in the paired analysis (a total of 29 ESTs) were predominantly young (mean age 31 ± 13 years), with 50% male. Most patients were also on beta-blockers (80%); in prior stress tests conducted before flecainide initiation, one patient had been trialled on amiodarone and another on sotalol. Consistent with institutional protocol, no beta-blocker or anti-arrhythmic drug washouts were performed prior to the exercise stress tests. Apart from flecainide, background medical therapy remained unchanged between the off- and on-flecainide exercise stress tests in all patients.

We observed a significant reduction in ventricular ectopy following flecainide initiation (*Figure 1*). PVC frequency during exercise decreased from 3 ± 3 to 1 ± 2 PVCs/min (*P* = 0.023), and during recovery from 12 ± 13 to 2 ± 4 PVCs/min (*P* = 0.010). The prevalence of PVCs during exercise decreased from 86% to 47% (*P* = 0.027), and during recovery from 86% to 47% (*P* = 0.027).

**Figure 1:**
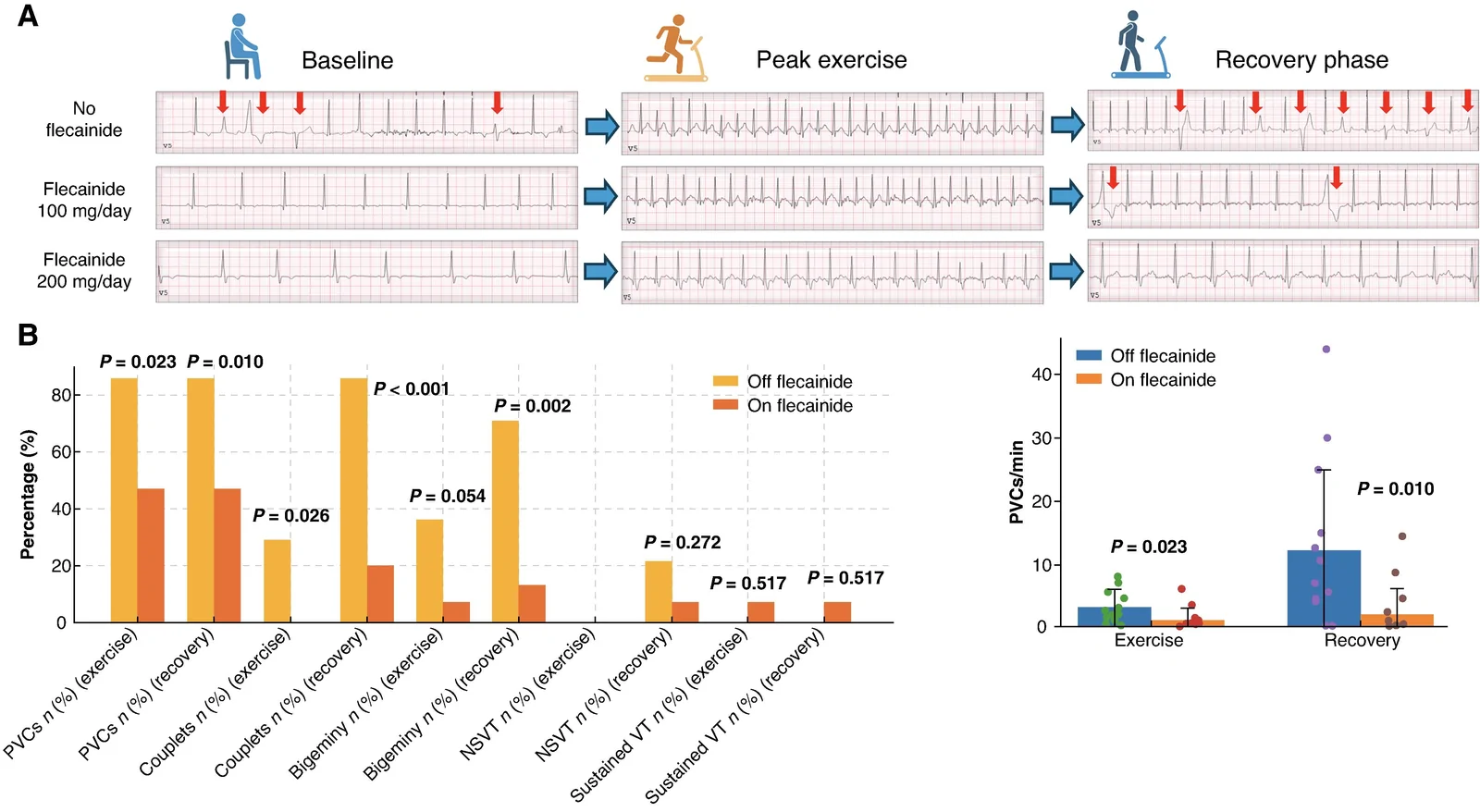
Flecainide reduces peak exercise and recovery phase ventricular arrhythmia in patients with PKP2-related arrhythmogenic cardiomyopathy. (*A*) Representative baseline, peak exercise and recovery phase ECG tracings in PKP2-ACM pre- and post-flecainide. (*B*) Comparison of pre- and post-flecainide arrhythmic burden during exercise stress testing (*n* = 29 ESTs).

Flecainide also significantly reduced more complex ventricular ectopy. Couplets during exercise were eliminated (29%–0%, *P* = 0.026), and during recovery were markedly reduced (86%–20%, *P* < 0.001). Bigeminy occurred less frequently both during exercise (36%–7%, *P* = 0.054) and recovery (71%–13%, *P* = 0.002). There were no significant differences in the frequency of NSVT or sustained VT during ESTs on vs. off flecainide.

Notably, five patients (63%) had a history of sustained VT prior to flecainide treatment. None of these individuals experienced breakthrough sustained arrhythmias during a median follow-up of 11 months after flecainide initiation. One patient without prior VT had an early sustained event shortly after starting flecainide but remained arrhythmia-free after dose adjustment. Patient-level analysis (one EST before and after flecainide) showed similar trends with reduced PVCs and significant reductions in recovery-phase couplets (*P* = 0.002) and bigeminy (*P* = 0.017); NSVT and sustained VT were not observed. The mean interval between paired off- and on-flecainide exercise stress tests was 8 ± 5 months.

These findings suggest that flecainide effectively suppresses exercise- and recovery-related ventricular ectopy and complex arrhythmias in PKP2-related ACM. The most pronounced benefit was during the recovery phase, a potential emerging marker of arrhythmic vulnerability in this population. Importantly, flecainide appeared safe in this small cohort, with no proarrhythmic events after dose stabilization.^6^ In addition to the observed reductions in ectopic burden, the lack of breakthrough arrhythmic events among previously symptomatic patients suggests that flecainide may provide a protective effect against VA in PKP2-ACM.

This aligns with mechanistic studies showing that flecainide not only stabilizes sodium channel–mediated conduction abnormalities associated with desmosomal dysfunction but also suppresses triggered activity through its effect on RyR2.^7^ The favourable safety profile in our cohort challenges historical contraindications based on ischaemic cardiomyopathy data and builds on findings from the recent multicentre study, supporting broader reassessment of safety assumptions.^5,8^

Importantly, this study contributes to the growing body of literature suggesting that not all ACM genotypes should be approached uniformly. The phenotype of PKP2-ACM appears uniquely sensitive to adrenergic stimulation and mechanical stress, with recovery-phase arrhythmogenesis serving as a genotype-specific biomarker of electrical instability. As such, individualized arrhythmia monitoring using tools like EST could play a pivotal role in both risk stratification and therapeutic monitoring.

This study has several limitations, including the small sample size, potential confounding from background beta-blocker therapy, inability to perform dose stratification, and the interval between paired ESTs, which may have contributed to spontaneous variability in arrhythmic burden; therefore, these findings should be considered hypothesis-generating. Given the parallels with CPVT, where exercise testing is used both diagnostically and therapeutically to guide flecainide titration, our data support the exploration of EST and flecainide response as a functional tool in PKP2-ACM management. Prospective studies are warranted to evaluate whether the reduction in EST arrhythmias translates into improved long-term outcomes and to assess the potential of exercise-guided flecainide dosing strategies

## Data Availability

The data underlying this article cannot be shared publicly to protect the privacy of the individuals that participated in the study. The data will be shared on reasonable request to the corresponding author.
